# Neurotoxins Acting at Synaptic Sites: A Brief Review on Mechanisms and Clinical Applications

**DOI:** 10.3390/toxins15010018

**Published:** 2022-12-27

**Authors:** Kunming Zhou, Weifeng Luo, Tong Liu, Yong Ni, Zhenghong Qin

**Affiliations:** 1 Department of Pharmacology and Laboratory of Aging and Nervous Diseases, College of Pharmaceutical Sciences, Suzhou Medical College of Soochow University, Suzhou 215123, China; 2 Department of Neurology and Clinical Research Center of Neurological Disease, The Second Affiliated Hospital of Soochow University, Suzhou 215004, China; 3 Institute of Pain Medicine and Special Environmental Medicine, Nantong University, Nantong 226019, China

**Keywords:** neurotoxins, synapse, botulinum toxin, cobrotoxin, pain

## Abstract

Neurotoxins generally inhibit or promote the release of neurotransmitters or bind to receptors that are located in the pre- or post-synaptic membranes, thereby affecting physiological functions of synapses and affecting biological processes. With more and more research on the toxins of various origins, many neurotoxins are now widely used in clinical treatment and have demonstrated good therapeutic outcomes. This review summarizes the structural properties and potential pharmacological effects of neurotoxins acting on different components of the synapse, as well as their important clinical applications, thus could be a useful reference for researchers and clinicians in the study of neurotoxins.

## 1. Introduction

There are thousands of biological species that can produce toxins, and most of them are neurotoxins [1]. Over millions of years of evolution, biological toxins have acquired specific selectivity to interfere with some physiological functions and disrupt a large number of basic neurobiological processes such as synaptic transmission [2].

At the neuromuscular junction (NMJ), nerve signals from spinal motor neurons are transmitted to the muscles via the release of synaptic acetylcholine (Ach), which causes muscle contraction. Structurally, there are three main components of the NMJ: the presynaptic nerve terminals, the synaptic cleft, and the postsynaptic receptors, mainly the dense cluster of nicotinic acetylcholine receptors (nAChRs) [3]. Ach-containing synaptic vesicles at presynaptic nerve terminals initiate the process of coalescing with the presynaptic membrane; then, the nerve terminal depolarizes, leading Ca^2+^ to flow in through voltage-gated channels. As a result of the influx of Ca^2+^, the vesicles begin to fuse with the membrane surface. Subsequently, Ach is released to the synaptic cleft from vesicles [4]. The Ca^2+^-mediated exocytosis of Ach described above primarily depends on the SNARE (soluble N-ethylmaleimide-sensitive factor attachment protein receptor) complex assembly [5], which generally comprises syntaxin, VAMP, and SNAP-25 homologs. The AChR is a type of ligand-gated ion channel [6] formed by five subunits, and it has several subtypes and these subtypes are assembed of 17 different subunits (α1–α10, β1–β4, γ, δ, and ε) [7]. The different AchRs are expressed in specific regions of the brain and peripheral tissues of mammals [8]. The AchR was first identified as a classic neurotransmitter receptor [9], and recently there is also evidence showing that the nAChR exists in non-neuronal cells, including certain types of tumor cells. When it is activated, it promotes the development of tumors by inducing the release of autocrine growth factors [10].

Generally, the release of ACh and the process of Ach binding to the AchR are two of the main targets of neurotoxins [4]. Many neurotoxins are presynaptic neurotoxins that act at the presynapse and specifically bind to ion channels to regulate neurotransmitter release or to block the neuronal transmission of signals [1]. According to this feature, presynaptic neurotoxins can be roughly divided into four categories: (1) clostridial neurotoxins that block the neurotransmitter function by their specific metalloproteolytic activity aiming at SNARE proteins; (2) presynaptic neurotoxins in snake venoms with phospholipase A2 activity; (3) excitatory latrotoxin-like neurotoxins [11]; and (4) neurotoxins that function via various ion channels (such as calcium channels, potassium channels, etc.) [12]. There are also a variety of neurotoxins that have postsynaptic effects. They bind to acetylcholine receptors, acetylcholinesterase, or ion channels, thereby altering cholinergic functions [1]. There are a large class of snake neurotoxins defined as postsynaptic neurotoxins, collectively called “α-neurotoxins” [13]. All members of the α-neurotoxin family show high similarity in the mechanism of neuromuscular paralysis, in which they primarily target and then bind the postsynaptic nAChRs, specifically at the NMJ [14,15,16].

This review aims to discuss the structures and mechanisms of action of the presynaptic and postsynaptic neurotoxins and to provide a summary of recent developments in clinical applications of these neurotoxins. This review might be considered as a first-hand reference for experts and clinicians interested in translating these neurotoxins into clinical therapies and uncovering the underlying mechanisms associated with clinical efficacy. Critical comments on the prospects for clinical application of these neurotoxins in diverse diseases are also provided.

## 2. Presynaptic Neurotoxins

### 2.1. The Clostridial Neurotoxins

**Figure 1:**
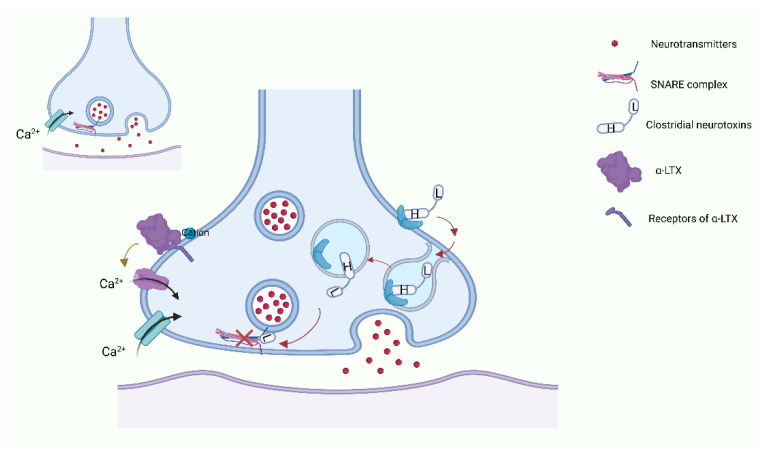
This is a synaptic structure. The top left is the normal release of neurotransmitters from synapses in response to calcium stimulation; in the middle, botulinum toxin and α-LTX affect neurotransmitter release by acting on synapses (created with BioRender.com, accessed on 20 December 2022). SNARE: soluble N-ethylmaleimide-sensitive factor attachment protein receptor.

The anaerobic bacteria that belong to the clostridia class are a vital threat to human health, causing a variety of disorders such as food poisoning and, even worse, gas gangrene [17]. Above all, two main categories of clostridial neurotoxins are considered as the most potent toxins that we have known: botulinum neurotoxins (BoNTs) and tetanus toxins (TeNTs) [18]. These two types of toxins have a similar molecular structure: 150 kDa molecular size with three main functional domains [18], the light chain (LC, 50 kDa) and the heavy chain (HC, ~100 kDa) with two domains. The LC and HC are connected by a disulfide bond. Both BoNT and TeNT act in a similar way: (i) binding to the presynaptic membrane; (ii) internalization; (iii) membrane translocation and release of the LC into the cytosol; and (iv) cleavage of SNAREs driven by the LC [19]. The HC will form a channel to help translocation of the LC into the cytosol [20]. The C-terminal part of the HC mediates the interaction of the toxins with neurons [21] via a neurospecific double binding to the polysialoganglioside and the glycosylated lumenal domain of a synaptic vesicle protein, which then leads to the internalization of the toxin [18,19]. The function of the N-terminal part of the HC is not fully understood, although some studies revealed that it protects the LC from the cutting of non-specific substrates until the LC is localized within the cytosol [22,23]. The LC is a metalloprotease [24], which has selectivity for SNARE proteins and can cut SNARE at different peptide bonds [25]. This specific structure is the basis for the action of BoNT and TeNT [26]. Both toxins can target and enter the nerve terminals at NMJs, then change the conformation to enable translocation of the LC into the cytosol. In there, the release of the neurotransmitters is disturbed by the LC via cleaving SNARE proteins [27,28,29] (Figure 1).

The neuroselectivity of BoNT and TeNT is probably due to the following aspects: (1) The C-terminal part of the HC mediates the interaction of the toxins with their receptors, and the receptors are mainly enriched in the neuronal terminals. (2) The receptor-mediated endocytosis makes them enter the neuronal cells, but BoNT and TeNT enter in different endocytic vesicles. (3) SNAREs, the target molecules of BoNT and TeNT, are expressed in the neuronal cells of almost all vertebrate phylla [30,31,32]. However, the neuroselectivity of BoNT is not absolute and it can act on the non-neuronal cells to exert some functions, such as glial cells [33].

Up to now, there are eight BoNT serotypes that have been identified and named as A–G and X. They are classified due to lack of cross-neutralization by different antisera against each toxin type [34,35]. Over 40 subtypes have been identified [36]. They bind to different receptors to drive the process of internalization [19]. The flaccid paralysis induced by BoNTs occurs primarily due to the blockade of peripheral cholinergic nerve endings [21,37,38,39], whereas TeNT, produced by a Gram-positive bacillus, *Clostridium tetani* [40], has only one type [18]. The release of neurotransmitters, such as GABA and glycine, is blocked by TeNT which can lead to spastic paralysis [41,42,43]. With prolonged action, it causes death when muscular hypertonus occurs in the respiratory muscle and leads to breathing failure [44].

The two clostridium neurotoxins act in a similar way, but they cause very different diseases. The reason for this is that TeNT travels retroaxonally and is transferred via a trans-synaptic movement to inhibitory interneurons in the CNS to block the release of neurotransmitters, which results in motor neuron hyperactivity and spastic paralysis. However, BoNT mainly acts on the NMJs to inhibit the release of acetylcholine and then induce flaccid paralysis [18,41,45]. Specifically, TeNT moves retroaxonally along the axons of motor neurons into the cell body, releases and thereby enters the connecting inhibitor neurons, and then the LC exerts the function of blocking neurotransmitter release [46]. TeNT can bind to not only the connecting inhibitor neurons but also the dendrites of sensory and adrenergic neurons [41].

### 2.2. Excitatory Latrotoxin-like Neurotoxins

There are high-molecular neurotoxins extracted from the venom of black widow spiders called latrotoxin-like neurotoxins (LaTXs). They consist of various specific types: one vertebrate-specific toxin (α-latrotoxin (α-LTX)) [47], five highly specific insecticidal toxins (α-, β-, γ-, δ-, and ε-latroinsectotoxin (LITs)) [48], and one crustacean-specific toxin (α-latrocrustatoxin (α-LCT)) [49]. LaTXs are secreted into the gland lumen as 160 kDa inactive precursor polypeptides. In the gland lumen, the N-terminal signal peptide and a C-terminal inhibitory domain are cleaved and proteolyzed, which produces the final mature 130 kDa toxin [39,40].

Among the above-mentioned toxins, there are a number of studies on α-LTX. α-LTX causes a syndrome called lactrodectism in the clinic, which has the feature of serious muscle spasm and lots of other effects, for example, hypertension, sweating, and vomiting [50,51]. The α-LTX also affects the process of exocytosis and has a high affinity for three types of receptors: cell adhesion protein neurexin (NRX) [52,53,54]; G-protein-coupled receptor latrophilin (LPHN or CIRL) [55,56]; and the receptor-like protein tyrosine phosphatase σ (PTPσ) [57]. The α-LTX initiates the release of neurotransmitters by two distinct mechanisms, both of them relying on the binding of the toxin to three types of receptors [58,59,60]: (1) in a Ca^2+^-dependent manner: α-LTX binds to the cell adhesion protein neurexin in the presence of Ca^2+^ and then inserts into the plasma membrane to form the pore and thereby induces the influx of Ca^2+^ [61], and (2) in a Ca^2+^-independent manner: it binds to the other two receptors without Ca^2+^. Furthermore, LPHN may mediate the process of stimulating PLC, producing IP3 and diacyl glycerol, releasing the stored Ca^2+^, and activating PKC. This cascade promotes the release of neurotransmitters form vesicles [60,62] (Figure 1).

### 2.3. Presynaptic Neurotoxins from Snakes

Most snake venoms contain both pre- and postsynaptic neurotoxins [63], whereas some snake venoms contain only presynaptic neurotoxins [64]. These presynaptic neurotoxins belong to phospholipases A_2_ (PLA_2_), which are Ca^2+^-dependent enzymes [63]. They can hydrolyze the sn-2 ester bond of 1,2-diacyl-3-sn-phosphoglycerides to produce fatty acids and lysophospholipids [11,65]. Various snake presynaptic PLA_2_ neurotoxins have a similar secondary structure with three larger α-helices and a short two-stranded β-sheet [66,67]. The pharmacological effects of these neurotoxins are also attributed to their PLA_2_ enzymatic activity, including antibacterial, cardiotoxic, and neurotoxic actions [68,69,70,71,72,73,74,75].

The studies on PLA_2_ snake neurotoxins are extensive, and the neurotoxicity induced by them can be attributed to the inhibition of presynaptic neuromuscular transmission [76]. Snake presynaptic neurotoxins exert a function that blocks the release of ACh in the nerve terminals [63] because they can strongly reduce the frequency of spontaneous exocytosis that releases ACh from small synaptic vesicles (SSVs) but does not affect the amount of ACh in one single SSV [65]. The electron microscopic studies of NMJ affected by snake presynaptic neurotoxins revealed: (1) swollen and enlarged axon terminals; and (2) the emergence of lots of Ω-shaped plasma membrane invaginations [77,78,79,80,81]. Recently, a PLA_2_ neurotoxin MiDCA1 extracted from the venom of *Micrurus dumerilii carinicauda* coral snake [82,83] has been found to affect the process of the release of neurotransmitters [82] through targeting the Kv2 channels [84]. Thus, it was proposed that MiDCA1 blocks the Kv2.1 channels and thereby decreases the release of Ach [84].

### 2.4. Other Presynaptic Neurotoxins Acting on Ion Channels

The release of neurotransmitters, which happens in a Ca^2+^-dependent manner [85], relies on the voltage-gated Ca^2+^ (Ca_v_) channels (VGCCs) that have selective permeability to Ca^2+^ and can drive calcium influx [86,87]. Among all types of VGCCs, Ca_v_2 channels mainly regulate exocytosis. Ca_v_2 channels contain Ca_v_2.1(P/Q-type), Ca_v_2.2 (N-type), and Ca_v_2.3 (R-type). Furthermore, the release of neurotransmitters at ribbon synapses in the retina and inner ear mainly relies on Ca_v_1 channels (L-type) [88]. In addition to the above-mentioned types of presynaptic neurotoxins, there are some presynaptic neurotoxins that act on ion channels, such as agatoxins, conotoxins, and dendrotoxins.

Agatoxins are a class of toxins extracted from the American funnel web spider *A. aperta* that can target different types of ion channels. They have various components that are divided into three classes: α-Agatoxins, µ-Agatoxins, and ω-Agatoxins, according to their functions on different channels [89,90]. Among these, ω-Agatoxins are particularly special because they are selective for different subtypes of calcium channels [89,91]. The ω-Agatoxin IA, which has five disulfide bonds [92], appears to be selective for insect calcium channels; the ω-Agatoxin IIA with three disulfide bonds disturbs both mammalian and invertebrate calcium channels [93]; ω-Agatoxin IIIA with six disulfide bonds [93] acts as a pore blocker due to the poor selectivity among the family of high-voltage-activated channels [94,95]; and ω-Agatoxin IVA exerts functions as a gating modulator via interfering with the domains of the voltage sensor [96,97,98].

Conotoxins are peptides with distinct cysteine frameworks, and they mainly target various ion channels and receptors [99], such as G-protein-coupled receptors (GPCRs), transporters, and enzymes [100,101]. Among the various subgroups of conotoxins, ω-conotoxins, consisting of 24–30 amino acids [102], physically block the pore of the channel to prevent calcium influx [98,103,104]. The two most characterized ω-conotoxins are GVIA [105] and MVIIA [106], which can selectively inhibit the N-type VGCC (neuron-type voltage-gated calcium channel). Furthermore, the selectivity of MVIIA on the channel is weaker than that of GVIA, but it can dissociate quicker due to the weaker selectivity [107,108], and therefore it was developed as a therapeutic peptide ziconotide (Prialt^®^) for pain treatment [90].

## 3. Postsynaptic Neurotoxins

The postsynaptic neurotoxins have postsynaptic blocking functions at the skeletal muscle end-plate and neurons, named α-neurotoxins [15]. They act as antagonists of nAChRs in the NMJs and neurons [109]. The α-neurotoxin family has a similar mechanism of the function that causes muscular paralysis due to blockade of the postsynaptic nAChRs at the NMJ [110,111,112].

The α-neurotoxins in snake venoms belong to the three-finger toxins [113]. These kinds of toxins are flat molecules, and the reason for the name of “three-finger” is that the flat molecules have a small globular hydrophobic core, and this core can form three adjacent loops, with the shape of three fingers [111,114,115]. The three-finger toxins are mainly generated form Elapid snakes, including α-cobratoxin (α-Cbtx), α-cobrotoxin (α-Cotx), and α-bungarotoxin (α-Bgtx) [116]. In addition, based on their molecular structures, α-neurotoxins are roughly divided into three categories: short-chain toxins with 60–62 amino acids residues and four disulphide bridges (α-Cotx) [117]; long-chain toxins with 66–75 amino acid residues and five disulphide bridges (α-Cbtx) [118]; and weak toxins, which have the feature of hypotoxicity (LD50 ~ 5–80 mg/kg as compared to LD50 ~ 0.04–0.3 mg/kg for other toxins) [103]. What they have in common is that they all target muscle α1 nAChRs, but only long-chain α-neurotoxins can bind to the α7 nAChR with high affinity [109,119,120], while only the dimmer of cobrotoxin binds to it. As we know, the α7-type nAChR is considered as a potentially essential target for the treatment of many diseases in the clinic [121], such as cognitive disorders [122,123], inflammatory diseases, and chronic pain [124]. Alzheimer‘s disease, Parkinson’s disease, Lewy body dementia, and schizophrenia all have the feature of neuroinflammation [125,126,127,128,129,130,131,132,133]. At present, some researchers have started to explore the effect of α-neurotoxins on central nervous system diseases [134,135,136,137,138]. Among these toxins, the α-bungarotoxin (αBgt) is the most popular one as a pharmacological tool used for studying α7 nAChR [121] because of the practically irreversible binding to the Torpedo acetylcholine receptors [139].

**Figure 2:**
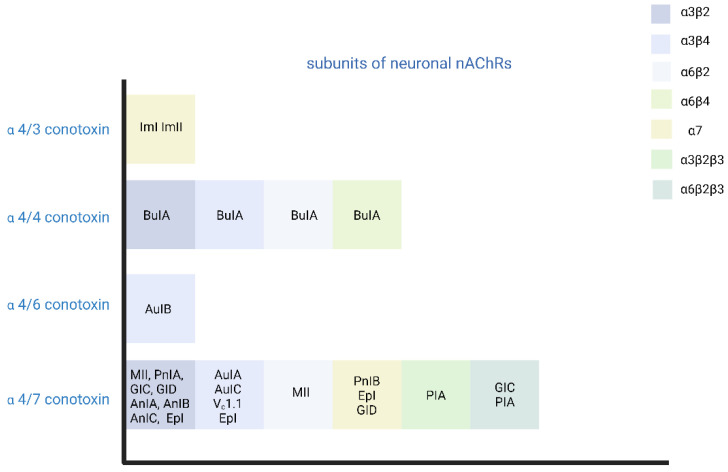
Different subgroups of α-conotoxins are selective for different subtypes of AChRs. (Created with BioRender.com, accessed on 29 October 2022).

In addition to snake neurotoxins, α-conotoxins generated from marine organisms of the genus Colocasia also target nAChRs [140]. There are many subgroups of α-conotoxins that are selective for different subtypes of AChRs, such as α3/5-conotoxins that selectively block the muscle nAChR, and α4/3-, α4/4-, α4/5-, α4/6-, and α4/7-conotoxins that selectively block the neuronal nAChRs [140,141,142] (Figure 2).

All the above-mentioned neurotoxins that are classified into presynaptic and postsynaptic neurotoxins, even though they are from different sources, have many types, and their complex subtypes and structures make it difficult to generalize with a symbolic model that they have a similar principle of function in the intervention of the neuromuscular signal transmission process, in which they affect the transmission of neurotransmitters at synaptic sites by regulating ions, receptors, and membrane fusion proteins and in other ways.

Besides those mentioned above, there are still some toxins not marked as “neurotoxins” that exert the same action on the nervous system. For example, the epsilon toxin (ET), produced by clostridium perfringens types B and D, can cause enterotoxaemia in sheep, goats, and cattle. When it enters the brain, it targets the neural cells and also induces the release of glutamate and other transmitters; but the specific mechanism needs more exploration [143]. Some bacterial enterotoxins, such as toxin B (TcdB) and cholera toxin (CT), can attack the enteric nervous/endocrine system. There is evidence that CT activates a secretomotor neural reflex to further enhance the secretory responses; and TcdB can catalyze the glucosylation of Rho proteins (Rho, Rac, and Cdc42) to block neurotransmitter exocytosis [30,144,145,146,147].

## 4. Clinical Applications of Neurotoxins

For the clinical applications of neurotoxins, besides their pharmacological effects, we also need to consider their half-life in vivo and their distributions in our body to exert their clinical effects. For most neurotoxins, they are limited to the injection compartment and cannot cross the barrier due to their peptide structure [148]. However, there are some special toxins that can break through this limitation, such as apamin (a peptide found in bee venom), which has the capacity to cross the blood–brain barrier [149]. Moreover, (1) toxins impairing the blood–brain barrier, such as clostridium perfringens epsilon toxin, can cross the barrier [143]; and (2) neurotoxins with retrograde axonal transport can reach the target, such as TeNT, mentioned above [46]. Because of this feature, they can be a more effective treatment in pain management [150].

### 4.1. Clostridial Neurotoxins

As we can see from Table 1, only BoNT/A and BoNT/B have been officially approved. The use of BoNT/B is limited due to its higher incidence of adverse effects than that of BoNT/A [151,152]. In addition, it does not have such long-lasting effects as BoNT/A [153]. The current treatments with BoNT are mainly used in the area of dystonic muscle contractions, which depends on the inhibition of the excessive release of the ACh neurotransmitter [154]. Recently, several articles also revealed that for some other diseases, such as chronic pain that is associated with a variety of neurological disorders (trigeminal neuralgia), neuroinflammation [155,156], depression [157], and skin diseases [158], BoNT can also play a therapeutic role. However, the therapeutic effects of BoNT mentioned above cannot be simply explained by the mechanism of NMJ blockade and needs more exploration. It has a long half-life in vivo, and one of the factors responsible for this phenomenon is that the catalytic light chain escapes from the proteasomal degradation by binding to the deubiquitinating enzyme, VCIP135/VCPIP1, and therefore remains enzymatically active for months [159,160]. This explains the clinical phenomenon that after the injection of BoNT in the detrusor muscle, the patients with neurogenic urinary incontinence do not need additional injections for 36 weeks and side effects are not observed [161,162]. For its distribution, sometimes it needs to cross barriers to reach the target area, and there is evidence that it can travel in a way similar to TeNT [18,163]. Therefore, it can influence the ascending pain processing pathway [150].

Owing to the technical advances in recombinant DNA technology and purification techniques of recombinant proteins, more and more engineered BoNTs are produced for clinical uses [184]. The indications of them are classified into the following categories based on the inhibition of different transmitters or other unclear mechanisms. For the inhibition of Ach release, here are the indications:

#### 4.1.1. Dystonic Muscle Contractions

From a translational perspective, when the nerve–muscle impulse is inhibited, NMJ function can be restored after BoNT treatment, which provides a scientific basis for BoNT in the treatment of various human diseases characterized by hyperfunction of cholinergic activity [1]. The initial clinical application was the treatment of benign essential blepharospasm by Scott and his coworkers [185]. Therefore, BoNT was first applied in the clinic for patients with dystonia and had a remarkable benefit, and it is now still considered as a choice of treatment for patients with problems of focal or segmental dystonia, including blepharospasm, oromandibular dystonia, spastic dysphonia, and so on. Furthermore, the treatment of hemifacial spasm and primary dystonia, such as cervical dystonia, is another application of BoNT in the clinic. What is more, BoNT can also be used for the treatment of some occupational dystonias, such as writer’s and musician’s cramps and stroke-related hemiplegia [186]. For laryngeal dystonia, characterized by spasmodic dysphonia, which manifests as either a laborious and tense sound that is interrupted by frequent articulatory interruptions and silent pauses, or a respiratory murmur, BoNT is also considered the treatment choice [187]. As for gastrointestinal, genitourinary, and sphincter disorders (such as achalasia, spasm of the sphincter of Oddi, and anal fissure [186]), the therapeutic efficacy of BoNT also acts on the spasm muscles in these organs [162,188,189,190,191]. Generally speaking, BoNT mainly leads to the alleviation of dystonic muscle contractions.

BoNT also interferes with transmission located at the cholinergic autonomic parasympathetic and postganglionic sympathetic nervous system, and therefore this toxin has been widely used for the diseases of the autonomic nervous system [192], such as essential focal hyperhidrosis, which is characterized by excessive sweating of the palms, feet, or axillae, and it is related to secretomotor hyperactivity [192]. After the treatment with BoNT, the patients’ quality of life is significantly improved due to a significant improvement in the symptoms [193,194,195]. The current clinical applications of botulinum toxin are mainly concentrated in the field of aesthetic medicine, such as the glabellar frown lines [196] and aging neck (hypertrophic platysma muscle bands) [197].

#### 4.1.2. Skin Diseases

For skin diseases, BoNTs have some label and off-label applications [158]. In sweat gland disorders (idiopathic hyperhidrosis, chromhidrosis, and bromhidrosis), the patients mainly suffer from excessive sweating in one or more parts of the body. The BoNT injection can decrease sweat secretion [198] by preventing the release of Ach and some other neurotransmitters from presynaptic vesicles [199]. In alopecia (alopecia areata and androgenetic alopecia), a study showed that BoNT downregulated the expression of TGF-β, thus suppressed follicular epithelial cell growth [200].

#### 4.1.3. Neuropathic Pain and Neuroinflammation

BoNT can inhibit not only the release of Ach but also other neurotransmitters [32]. For several types of neuropathic pain such as trigeminal, posttraumatic, or postherpetic neuralgia, significant analgesic effects have been observed after administration of botulinum toxin A (BoNT/A) [165,201,202]. It is speculated that BoNT/A exerts its therapeutic effect by inhibiting the process of the secretion of some pain mediators (substance P, glutamate, and calcitonin-gene-related protein (CGRP)) and other pain transmitters released from the nerve terminals and dorsal root ganglions (DRGs) and trigeminal sensory neurons [165,203,204,205]. There is evidence that after peripheral administration of BoNT/A, the antinociceptive action is not primarily mediated by the direct prevention of central CGRP release. This indicates that it might depend on the toxin’s axonal transport [206].

Research revealed that intraplantar administration of BoNT/A alleviated mechanical and thermal hypersensitivity and the activation of microglia induced by chronic constriction nerve injury in the ipsilateral lumbar spinal cord in rats [207], decreased the amount of the pro-inflammatory cytokines IL-1β and IL-18, and increased the level of IL-1 receptor antagonist and IL-10 in the spinal cord and/or the DRG [167]. Recently, an in vitro study indicated that in primary rat microglia the expression of pro-inflammatory IL-1β, IL-18, IL-6, and nitric oxide synthase 2 (NOS2) was inhibited by BoNT/A, and the intracellular signaling pathways mediated by p38, ERK1/2, and NF-κB were also inhibited. Additionally, the expression of SNAP-23 was decreased, whereas the expression of TLR2 was increased [208].

#### 4.1.4. Depression

The first article that reported the antidepressant effect of BoNT was in 2006. It was found that the self-rated depression scores on the Beck Depression Inventory (BDI) II were significantly improved after 8 weeks in ten middle-aged women that had moderate-to-severe, partly chronic, and treatment-resistant depression when they received one single application of BoNT administration in the glabella [209]. Subsequently, several randomized controlled trials were conducted, and they confirmed the efficacy of BoNT for the treatment of depression [210,211,212,213,214]. With more and more evidence showing that BoNT can be used for the treatment of depression, it is important to find the underlying mechanisms of this action. So far, several possible mechanisms have been proposed. Firstly, there is a feedback loop from the face to the brain, called “emotional proprioception”, that can reinforce and maintain the negative emotions. When BoNT/A disturbs it, depression can be ameliorated [215]; Secondly, in rat depression models, there is evidence that after the administration of BoNT in the face, the metabolism of monoaminergic neurotransmitters is changed in the limbic brain regions [216]. Thirdly, some experimental evidence shows that high-dose [217], locally injected BoNT can enter the central nervous system (CNS), possibly through retrotransportation, which might be another mechanism for BoNT in regulating mood [218]. Moreover, some substances might be related to the mechanism of BoNT for antidepression, such as the RAS-related C3 botulinum toxin substrate 1 (Rac1). This is the central nervous substrate of BoNT [219]. Emerging evidence has also shown that the expression of BDNF diminishes in animal models of depression and depressed patients [220,221,222]. BDNF is essential for neurogenesis and the reduction in depression-like behaviors [223]. It promotes the phosphorylation of CREB through ERK activation [224]. A recent article has shown that the expression of BDNF at both the mRNA and protein level was up-regulated in the hippocampus by BoNT/A, and therefore the downstream ERK-CREB signaling pathway was activated in depression mice models [168].

#### 4.1.5. Headache

Headache is a common neurological disorder. According to the international classification of headache disorder (ICHD-3), it is classified into primary headaches and secondary headaches. Primary headaches include migraine, trigeminal autonomic cephalalgia (TAC), and tension-type headache (TTH). Secondary headaches include neuropathies, facial pains, and other forms of headache [225]. They all share the common pathophysiology of the abnormal activation of the trigeminovascular system [226], which leads to vasodilation and neurogenic inflammation and pain sensitization in the peripheral and central nervous system, resulting in the persistent headache [166]. The onabotulinum toxin A, as a BoNT formulation, has been used for the treatment of chronic migraine [169]. Several articles suggested that BoNT/A could suppress the release of neuropeptides and neurotransmitters that contain CGRP [227], substance P [228], and glutamate from sensory peptidergic sensory neurons [229]. In addition, BoNT/A can also interfere with the pain-sensing receptors expressed on the plasma membrane, including transient receptor potential cation channel vanilloid subfamily member 1 (TRPV1), transient receptor potential cation channel ankyrin subfamily member 1 (TRPA1), ATP-gated P2X receptor cation channel family 3 (P2X3) [230], and AMPA receptor [231,232,233,234]. All mechanisms mentioned above may contribute to the antinociceptive action of BoNT/A.

As for TeNT, it is the only known toxin that has the potential for selective improvement of motor functions, so the flabby and weak muscles induced by the injury of the brain and spinal cord can be treated with TeNT [170,235,236]. Due to the presence of the blood–brain barrier [2], it makes it difficult for some drugs to enter the brain. Another clinically relevant area of TeNT has generated great interest, which is its potential as a fusion protein or carrier to deliver drugs into the CNS. TTFC (a segment with 50 kDa from the carboxy-terminal HC of TeNT) can exert the function of delivery by interacting with neural gangliosides and specific proteins linked with lipid microdomains of the neuronal surface [171,237,238,239,240,241] and then moving retroaxonally to the CNS [242,243,244]. It can bind to polysialylgangliosides G_D_1b and G_T_1b and then is internalized by motor neurons at the NMJ. Finally, it reaches and influences the CNS via traveling retroaxonally [245,246]. Some potential therapeutic molecules, such as cardiotrophin-1 (CT1) and Bcl-xL, have been successfully transported into neurons by coupling with TTFC [245]. Furthermore, when coupling with these molecules, TTFC still keeps its capacity of neuronal binding [246]. However, their effects as therapeutic agents will need more studies in vivo.

Moreover, TTFC has also been demonstrated to have neuroprotective activities [246]. In the amyotrophic lateral sclerosis mice model, TIFC can modulate the levels of NLRP3 and caspase-1 in the spinal cord and reduce the level of IL-6 in tissues [247]. In the models of Parkinson’s disease (PD) and Alzheimer’s disease (AD), it can be used for neuronal dysfunction, learning impairment, and memory impairment [248,249]. TTFC can protect against apoptosis via activating the MAPK/ERK pathway to inhibit the disruption of the dopaminergic neurons caused by MPP^+^ [245].

There are several engineered BoNTs; here are some of them: (1) the Erythrina cristagalli lectin replaces the C-terminal of the HC of BoNT, and thereby forms a novel conjugate. It binds to eDRG neuronal cell types and inhibits the neurotransmitter release [250]. After injecting it into the intrathecal space of a mouse, it was demonstrated to have a long-lasting analgesic effect [251]; (2) the botulinum construct (Bitox), synthesized by “stapling” the recombinant LC/translocation domain of BoNT/A and receptor-binding domain has the potential to treat pain and does not result in muscle paralysis [252,253]. The recombinant LC/translocation domain of BoNT/A can bind to the TeNT receptor-binding domain [254]; (3) the chimeras of BoNT/A and BoNT/E can significantly reduce the acute nociception induced by capsaicin [255].

### 4.2. LaTXs

α-latrotoxin is an inducer of Ca^2+^ influx and is expected to ameliorate dysfunctions and diseases that are associated with the reduction in the release of transmitters and hormones. It is also expected to treat metabolic diseases, such as type I diabetes [172,256]. The structure of α-latrotoxin is homologous to glycogen-like peptide-1 (GLP1) an endogenous peptide, and it can control the level of blood glucose by binding to GLP1 receptors in the cell membrane of pancreatic β-cells to increase the release of insulin. Therefore, α-latrotoxin has been used as a potential drug for the treatment of obesity, diabetes, and other related metabolic disorders [257,258,259].

### 4.3. Snake Presynaptic Neurotoxins

#### 4.3.1. Anticancer

Snake presynaptic neurotoxins have PLA_2_ activity. PLA_2_ has been reported to have anticancer properties, which involve inhibiting angiogenesis, migration, and adhesion of various kinds of cancer cells [260]. For example, crotoxin extracted from the venom of South American rattlesnake has been found to have potential anticancer effects in several types of cancers [173]. Several experiments demonstrated that crotoxin arrested the cell cycle at the G_2_/M phase [261]. Furthermore, recently it has been reported that crotoxin is also a potential regulator of the signaling cascade involved in epithelial–mesenchymal transition (EMT) [262]. Crotoxin may have a high affinity for EGFR [263], which suggests that crotoxin might modulate the EGFR signaling pathway to exert its anti-tumor activity in SPCA-1 cells [264]. The research on lung cancer has attracted people’s attention because it is the leading cause of cancer-related mortality worldwide [265]. In human lung squamous carcinoma SK-MES-1 cells, crotoxin can induce apoptosis and autophagy through the p38MAPK signaling pathway in vitro [266]. Furthermore, in the process of treating cancer with crotoxin, a phase I clinical trial revealed that besides its anticancer effect, analgesic effects were also observed in terminal cancer patients [267]. In some other animal models, such as acute pain models, studies have demonstrated that crotoxin also showed the effect of antinociception [268] without the involvement of opioid receptors [175,269], instead of the central cholinergic, serotonergic, and noradrenergic systems [270].

#### 4.3.2. Antibacterial

In addition to the anticancer and analgesia effects, crotoxin also exerts an antibacterial effect because of the change in membrane permeability induced by PLA_2_, which destroys the integrity of the bacterial cell membrane [271].

### 4.4. Other Presynaptic Neurotoxins Acting on Ion Channels

#### 4.4.1. Analgesia

For presynaptic neurotoxins targeting calcium and potassium channels, the clinical applications mainly depend on their ability to block or activate the channels. For example, voltage-gated calcium channels are important for the process that conducts pain signals from the periphery into the dorsal horn of the spinal cord. Some conditions of pain that are difficult to treat with clinically available drugs, such as cancer and neuropathic pain, can be treated effectively with crotoxin [272].

Due to the selective blockage of the Ca^2+^ channel by ω-agatoxin, it may be potentially useful in clinical applications to treat a variety of disorders. Evidence from behavioral and electrophysiological reports suggests that ω-agatoxin IVA-sensitive P-type channels significantly modulate spinal nociceptive processes [176,273]. A study reported that intrathecal administration of ω-agatoxin IVA reduced late nociceptive behaviors induced by formalin [274].

Conotoxins have been widely used in basic neuroscience research to analyze neuronal voltage-gated Ca^2+^ currents, as well as in treatments in the clinic because of their powerful and pervasive ability to block Ca^2+^ channels. Conotoxins have the potential to become popular drugs [275,276] because ω-conotoxins specifically target presynaptic voltage-gated Ca^2+^ channels (VGCC), particularly N-type VGCCs, that have been proved to be involved in pain pathways, making ω-conotoxins potential analgesics [273]. Prialt™ (a form of the ω-conotoxin MVIIA) is a drug that has been approved by the FDA to treat the chronic pain that results from cancer- or AIDS-related neuropathy [177]. However, it is difficult for Prialt™ to cross the blood–brain barrier due to its inherent large molecular size and hydrophilicity [277], so its treatments are confined to intrathecal administration. Besides, there are various side effects (such as cognitive and neuropsychiatric adverse reactions [278]) that arise in the clinic and which need to be further investigated [273]. It is well known that the release of glutamate is essential in the process of spinal nociception, and the inhibition of the glutamate transporter GLT-1 can significantly reduce nociception behaviors [279]. Prialt™ is usually administered intrathecally to relieve pain by blocking VGCCs and abolishing capsaicin-evoked glutamate release in the spinal cord synaptosomes [220].

#### 4.4.2. Neuroprotection

Studies have reported that Prialt™ has neuroprotective potential after spinal cord injury (SCI), which suggests it may be a good alternative treatment for acute SCI [178]. After SCI, secondary neuronal death happens due to the glutamate-mediated excitotoxicity, leading to excessive intracellular calcium, mitochondrial dysfunction, acidosis, and the overproduction of free radicals [280,281,282,283,284,285,286]. This condition can be prevented by Prialt™ via inhibiting the release of glutamate [287,288] and calcium influx [276] and protecting mitochondria from traumatic brain injury [289,290,291]. Moreover, Prialt™ can reduce the expression of nNOS to inhibit apoptosis [178]. Furthermore, a recent article has reported a novel ω-conotoxin Bu8 with five amino acid residues and three disulfide bonds synthesized by Conus Bullatus. The potency in inhibiting N-type voltage-gated calcium (Ca_V_2.2) channels is twice that of MVIIA, and the inhibition of Ca_V_2.2 channels is highly selective, so there are fewer side effects [292].

Because dendrotoxins (DTXs) selectively bind to the Kv1.1 channel, they can be used to study the basic biology of K^+^ channels and the mechanisms of synaptic transmission. DTX plays an essential role in neuronal degeneration and seizures in nonclinical models. Deletion of Kcna1/Kv1.1 or Kcna2/Kv1.2 has been reported to cause epilepsy in rodents [179,180]. DTXs can bind to presynaptic K_V_ channels with a high affinity, which reveals their great potential to quantify the density of synapse in the CNS, and this potential usage might be applied to the diagnosis of neurodegenerative diseases that affect the integrity of the brain’s connectomes. For example, the loss of synapses in hippocampal tissue has been detected by α-DTX [2].

### 4.5. Postsynaptic Neurotoxins

Although there is currently no drug derived from α-neurotoxins on the market for muscle-associated diseases, there is data suggesting that some short-chain and long-chain α-neurotoxins that target the NMJ and other nAchRs have the potential therapeutic ability of treating disorders linked to NMJ dysfunction and others [181].

#### 4.5.1. Anticancer

One characteristic of many tumors is the increased expression of nAChRs. The enhancement of tumor cell proliferation and the acceleration of tumor growth are associated with nAChRs [293]. Ach, an autocrine growth factor of human lung cancer, can bind to nAChRs in lung cancer cells to accelerate their proliferation, migration, and invasion [293]. Studies have shown that the nicotinic and/or muscarinic receptors mediate the growth and apoptosis signals in mesothelioma and non-small-cell lung cancer (NSCLC) [294,295,296,297]. Furthermore, the activation of nAChRs stimulates lung cancer growth [294,298,299]. These effects suggest that α-neurotoxins have potential anticancer therapeutic prospects because they antagonize the receptors of the nAchRs. Moreover, α-cobratoxin has shown potential anticancer effects for NSCLC [182].

#### 4.5.2. Analgesia and Anti-Inflammation

We mainly discuss the long-chain toxin (cobratoxin) and short-chain toxin (cobrotoxin) in this review, focusing on their analgesic effects. Cobratoxin is purified from *Naja naja kaouthia*, and it has been suggested to have several essential functions including modulation of the nerves, suppression of the immune system, and anti-tumor, anti-inflammatory, and analgesic effects [183,300]. The possible analgesic mechanism is mediated by M_4_ mAChR, which is activated by cobratoxin and then triggers the CaMKII/CREB signaling pathway and inhibits low-potential Ca^2+^ channels [301]. Its effects of antinociception and anti-inflammation are also due to binding to the α7 subtype of nAChR with high affinity, which is followed by the decreased production of inflammatory cytokines such as TNF-α, IL-1, and IL-2 [302].

Cobrotoxin is purified from *Naja atra* venom (NNAV). It shows great inhibition of the inflammatory response in rat models of rheumatoid arthritis induced by adjuvant [303], as well as analgesic effects in rodent models of inflammatory pain induced by formalin and acetate [183]. It has been widely used for the treatment of chronic arthralgia, sciatica, and neuropathic headache in the clinic. Cobrotoxin is an approved drug by the CFDA [304] and has been called “Cobratide” in Chinese clinics. Recently, it has been reported that cobrotoxin in a higher dose has potential for the treatment of trigeminal neuralgia [305]. Cobrotoxin has similar analgesic action and underlying mechanisms of action as cobratoxin. It might have dual pain regulation, and there is a hypothesis to explain this phenomenon, namely, that cobrotoxin might activate the adenosine A_1_Rs and then inhibit the mitogen-activated protein kinases/extracellular signal-regulated protein kinase pathway to exert analgesic effects. However, with the increasing dose of cobrotoxin, the adenosine A_2A_Rs are activated to produce sensitization to pain [306]. Besides the analgesic effects, cobrotoxin can also exert the effect of anti-inflammation by interacting with the NF-κB signaling pathway [307]. The binding between cobrotoxin and the kinase domain of IKK can inhibit the phosphorylation of IKK and then prevent the release of free NF-κB from IκB, thereby reducing the transcription of inflammatory genes [308]. It is quite interesting that initial animal and clinical studies showed that cobrotoxin (Cobratide) can treat acute gout and relieve motor and some non-motor symptoms of PD (unpublished observations from Dr Qin’s lab).

## 5. Summary

Neurotoxins targeting the synapse structure have attracted attention for treating diseases that do not have a very effective therapy in the clinic. Up to now, natural neurotoxins have been proven to be highly useful for the treatment of many kinds of diseases, and the list of diseases being treated with neurotoxins keeps expanding. The neurotoxins that have been approved for clinical use by the FDA and CFDA are Botox, Xeomin, Dysport, Myobloc/Neurobloc, Cobratide, and Prialt™; the rest are currently under preclinical investigation for a variety of diseases.

### 5.1. Clinical Applications of FDA-Approved Neurotoxins

As we can see from Table 2, the clinical applications of neurotoxins, mainly BoNTs, on the market are still limited, and most of them focus on muscle-related diseases and pain. Therefore, other indications for these neurotoxins are now being studied to expand their range of applications. BoNT has been found to treat neuropathic pain, neuroinflammation, depression, and skin diseases, but the mechanisms involved have not been confirmed yet, which limits their use in the clinic.

### 5.2. Neurotoxins in Preclinical Studies

The other neurotoxins are mostly studied in the preclinical period. More information about their pharmacological properties and toxicity is needed. For example, our understanding of the Ca^2+^-independent effects of α-latrotoxin is still insufficient.

### 5.3. Expectations

In biological therapy, neurotoxic peptides offer great therapeutic potential, but they still have many functions that need to be discovered and validated. In this review, we briefly summarized the biological and clinical functions of neurotoxins aiming at synapses. There are methodological challenges, such as the fact that these neurotoxins are peptides and cannot cross the BBB or that they are unstable, which limits the wider use of neurotoxins and is a major challenge. However, these neurotoxins aiming at synapses have passed the test of time as biological therapeutics and have also made positive contributions in animal models. In the future, more and more detailed studies are needed, and the structure of these neurotoxins should be continuously improved to make them suitable for clinical applications and benefit human beings.

## Figures and Tables

**Table 1:** The summary of the clinical applications of neurotoxins (the “Indications” are found in the table).

Types of Neurotoxins	Trade Name (FDA Approved)	Indications (FDA Approved)		Clinical Applications	Reference
BoNT	Botox (BoNT/A) Xeomin (BoNT/A) Dysport (BoNT/A) Myobloc/Neurobloc (BoNT/B)	Botox	Overactive bladder (OAB) with symptoms of urge urinary; Urinary incontinence due to detrusor overactivity associated with a neurologic condition (e.g., spinal cord injury (SCI), multiple sclerosis (MS)) in adults who have an inadequate response to or are intolerant of anticholinergic medication; Neurogenic detrusor overactivity (NDO) in pediatric patients 5 years of age and older who have an inadequate response to or are intolerant of anticholinergic medication; Prophylaxis of headaches in adult patients with chronic migraine (≥15 days per month with headache lasting 4 h a day or longer); Spasticity in patients 2 years of age and older; Cervical dystonia in adult patients; Severe axillary hyperhidrosis that is inadequately managed by topical agents in adult patients; Blepharospasm associated with dystonia in patients 12 years of age and older; Strabismus in patients 12 years of age and older.	Dystonic muscle contractions Neuropathic pain Neuroinflammation Depression (under investigation) Skin diseases Headache	[164] [155,156,165,166] [167] [168] [158] [169]
		Xeomin	Chronic sialorrhea in patients 2 years of age and older; Upper limb spasticity in adults; Upper limb spasticity in pediatric patients 2 to 17 years of age, excluding spasticity caused by cerebral palsy; Cervical dystonia in adults; Blepharospasm in adults; Temporary improvement in the appearance of moderate-to-severe glabellar lines with corrugator and/or procerus muscle activity in adults.		
		Dysport	Cervical dystonia in adults; Temporary improvement in the appearance of moderate-to-severe glabellar lines associated with procerus and corrugator muscle activity in adults < 65 years of age; Spasticity in patients 2 years of age and older.		
		Myobloc/Neurobloc	Cervical dystonia to reduce the severity of abnormal head position and neck pain associated with cervical dystonia in adults; Chronic sialorrhea in adults.		
TeNT				Improve the motor functions Carrier to deliver into the CNS	[170] [171]
α-LTX				Type I diabetes (expected)	[172]
Snake Presynaptic Neurotoxins				Anticancer Antibacterial Antinociception	[173] [174] [175]
ω-agatoxin				Modulate the nociceptive process	[176]
Conotoxins	Prialt™ (Ziconitide) (a form of ω-conotoxin MVIIA)	Management of severe chronic pain in patients for whom intrathecal therapy is warranted and who are intolerant of or refractory to other treatment, such as systemic analgesics, adjunctive therapies, or intrathecal morphine.		Chronic pain (cancer- or AIDS-related neuropathy) Spinal cord injury	[177] [178]
DTX				Diagnosis of neurodegenerative diseases (potential)	[179] [180]
Postsynaptic Neurotoxins	Cobratide			Disorders linked to NMJ dysfunction Anticancer Anti-inflammation Analgesic effect	[181] [182] [183] [183]

**Table 2:** Summary of the clinical applications of the FDA-approved neurotoxins.

	Applications	
Botox	Muscle	Blepharospasm hemifacial spasm Strabismus cervical dystonia Upper limb and lower limb (adults) spasticity Bladder (neurogenic detrusor overactive (DO), overactive bladder (OB)) Forehead wrinkles
	Other	Migraine
Xeomin	Muscle	Cervical dystonia frown lines Blepharospasm upper limb spasticity
	Other	Sialorrhea in adults
Dysport	Muscle	Cervical dystonia Upper limb (adults) and lower limb (children + adults) spasticity Frown lines and wrinkles
Myobloc/Neurobloc	Cervical dystonia	
Prialt™	Severe chronic pain	
Cobratide	Chronic pain	

## Data Availability

Not applicable.
